# Electrospinning Mechanism of Nanofiber Yarn and Its Multiscale Wrapping Yarn

**DOI:** 10.3390/polym13183189

**Published:** 2021-09-20

**Authors:** Taohai Yan, Yajing Shi, Huimin Zhuang, Yu Lin, Dongdong Lu, Shengbin Cao, Lvtao Zhu

**Affiliations:** 1 Fujian Key Laboratory of Novel Functional Textile Fibers and Materials, Minjiang University, Fuzhou 350108, China; 2146@mju.edu.cn (Y.S.); 2563@mju.edu.cn (H.Z.); 2502@mju.edu.cn (Y.L.); 2 Key Lab for Sport Shoes Upper Materials of Fujian Province, Fujian Huafeng New Material Co., Ltd., Putian 351199, China; dongdong.lu@huafeng-cn.com; 3 School of Materials Science, Shanghai Dianji University, Shanghai 200003, China; caosb@sdju.edu.cn; 4 College of Textile Science and Engineering, Zhejiang Sci-Tech University, Hangzhou 310018, China; zhult@zstu.edu.cn

**Keywords:** electrospinning, nanofibers yarn, multiscale yarn, mechanical properties, mechanism

## Abstract

To analyze the feasibility of electrospinning nanofiber yarn using a wrapping yarn forming device, electrospun nanofiber-wrapped yarns and multiscale yarns were prepared by self-made equipment. The relationship between the surface morphology and properties of yarn and its preparation process was studied. The process parameters were adjusted, and it was found that some nanofibers formed Z-twisted yarns, while others showed exposed cores. To analyze the forming mechanism of electrospun nanofiber-wrapped yarn, the concept of winding displacement difference in the twisted yarn core *A* was introduced. The formation of nanofiber-wrapped structural yarns was discussed using three values of *A*. The starting point of each twist was the same position when *A* = 0 with a constant corner angle β. However, the oriented nanofiber broke or was pulled out from the gripping point when it was twisted, and it appeared disordered. The forming process of electrospun nanofiber-wrapped yarn displayed some unique phenomena, including the emission of directional nanofibers during collection, fiber non-continuity, and twist angle non-uniformity. The conclusions of this research have theoretical and practical value to guide the industrial preparation of nanofiber yarns and their wrapped yarns.

## 1. Background

Electrospun nanofiber materials have shown great application prospects in the field of functional textiles due to their ultra-fine fiber size, flexible material selectivity, structural controllability, and easy surface functionalization [1]; however, they generally have weak mechanical properties, and their nanofiber aggregates have poor structural stability, which severely restricts their practical applications as functional textiles [2,3]. In recent years, how to achieve the high-efficiency and controllable preparation of micro-nano fiber assemblies with stable structures, excellent mechanical properties, and long-term functions has become a popular research topic [4,5]. Yarns constructed based on electrospun nanofibers display the typical anisotropic structural characteristics of traditional yarns [6]. The fibers are aligned and closely contacted to form ultrafine capillary cavities and continuous channels [7]. This can overcome the inherent defects of the membrane structure nanofiber aggregates and has broad application prospects in biomedicine [8,9,10], smart wearables [11,12], sensing [13,14], functional textiles [15,16], and other fields, therefore promoting the industrial applications of electrospinning technology [17,18,19].

## 2. Introduction

Fiber and yarn are the main materials used in the traditional textile industry, but only fibers with a diameter larger than several microns can be processed using existing textile processing techniques [20]. Electrospinning can prepare fibers with diameters ranging from several nanometers to several micrometers; however, when the diameter reaches the nanometer level, the fracture strength and wear resistance of the fibers are relatively poor, making them difficult to process using traditional textile techniques [21]. According to the specific application requirements in textiles and apparel, microelectronic devices, and composite materials, it is necessary to form oriented nanofiber bundles and twisted yarns from them [22]. If nanofibers can be used in traditional textile methods such as knitting and weaving, yarns can be prepared from them [23]. This process involves collecting nanofibers into a certain orientation, followed by twisting the fiber bundles to improve the yarn strength and collective properties [24].

There are various methods for preparing nanofiber bundles with specific orientations. One is to use an auxiliary electrode method, including a spacer conductive plate collection device and a disk collection device to collect oriented nanofibers between parallel grounded double disks. This method is simple and effective, and the fiber orientation degree is high. The other method involves using the mutual attraction of oppositely charged fibers. Negative and positive spinning nozzles emit fibers with opposite charges to form fiber bundles at certain locations because of their charge attraction, which produces continuous oriented nanofibers; however, these fiber bundles are relatively fine [25]. Thirdly, continuous oriented nanofibers can be obtained by drawing oriented nanofibers with a zero-high-speed drum, but the degree of orientation is difficult to control, and the fibers are easily stretched and broken. There is another nanofiber collection method using a solution, in which fibers are collected into bundles, and the fibers within the bundles are oriented [26,27]. The fiber bundles are usually twisted mechanically or using airflow. The mechanical twisting method is highly controllable, simple, and reliable, and the yarn quality is good. This is the method used to produce most nanofiber yarns. Airflow twisting requires the use of professional nozzles and corresponding airflow twisting devices. The mechanism and process are relatively complex, and the twisting effect can change depending on the airflow parameters.

In contrast to electrospun pure nanofiber yarn, multiscale composite yarn is composed of traditional microfibers and electrospun nanofibers according to some structure rules. A nanofiber-wrapped core yarn or yarn wrapped on a core yarn may be a disordered or oriented nanofiber, and the oriented nanofiber is generally wrapped on the core yarn in a twisting manner. Zhou used electrospinning to collect nanofibers on parallel-aligned monofilaments and then aggregated and twisted monofilaments with nanofibers on the surface [28]. The nanofibers wrapped on the core yarn were disordered. Oriented nanofibers are mainly twisted by rotating hollow cylinders, disks, bell mouths, water bath vortexes, and air vortexes. Liu rotated a core yarn in the axial direction and collected oriented nanofibers between two parallel grounded aluminum sheets [29]. The nanofibers were twisted and wound on the surface to obtain an oriented wrapped yarn. Scardino used air vortex twisting to coat the surface of a core yarn with electrospun nanofibers. The prepared nanofiber-wrapped yarn also had a certain degree of orientation. Niu passed a core yarn through two hollow cylinders that were grounded relative to one another, and oriented electrospun nanofibers were collected between them [30]. One of the cylinders was used to twist the nanofibers on the core yarn, and the twisting orientation of the nanofibers was more apparent.

Multiscale yarn is made of a variety of fibers with different scales, which can fully integrate the structural characteristics and performance advantages of traditional microfibers and electrospun nanofibers. This allows them to make up for each other’s disadvantages while giving full play to the advantages of the textile yarn structure [31]. The weavability of nanofibers can be improved by preparing nanofiber yarns, and high-value-added functional textiles can be produced by combining the structural and functional advantages of conventional yarns and nano yarns. A composite yarn with this functional structure can be used without touching the surface of functional materials, such as fragrance finishing, energy storage, and temperature regulation. No studies have previously investigated the wrapping mechanism of nanofiber-wrapped yarns. In this paper, a self-made oriented nanofiber-wrapped yarn forming electrospinning device was designed. The nanofibers were twisted and wrapped on the core yarn by a disk. Electric field simulations were carried out on the device. The formation mechanism of the wrapped nanofiber structure was studied and verified.

## 3. Materials and Methods

### 3.1. Main Materials and Equipment

This process involves collecting nanofibers into a certain orientation, followed by twisting the fiber bundles to improve the yarn’s strength and collective properties. Polyacrylonitrile (PAN) was purchased from Taicang Kelda Plastic Materials Co., Ltd. (Taicang, China), with a molecular weight of 150,000. *N*, *N*-dimethylformamide (DMF) was purchased from Guangdong Jinhua Chemical Reagent Co., Ltd. (Guangzhou, China) and was analytically pure. 75D/36F twistless polyester multifilament was purchased from Fujian Jinjiang Technology Co., Ltd. (Jinjiang, China). High voltage DC power DW-P503 was purchased from Dongwen High Voltage Power (Tianjin, China) Co., Ltd. An LSP-10-18 micro-injection pump was purchased from Baoding Co., Ltd. (Baoding, China).

### 3.2. Experimental Method

#### 3.2.1. Self-Made Electrospinning Nanofiber Yarn Forming Device

**Figure 1:**
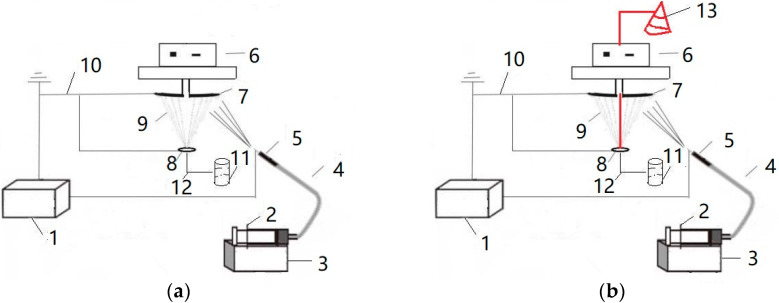
Schematic diagram of the forming device for electrospinning nanofiber yarn. (**a**) electrospinning nanofiber yarn forming device. (**b**) electrospinning nanofiber-wrapped yarn forming device. 1—High voltage DC power, 2—Syringe, 3—Micro-injection pump, 4—Infusion tube, 5—Needle tube, 6—Motor, 7—Receiving disk, 8—Ring, 9—Nanofiber, 10—Ground electrode, 11—Winding system, 12—Core yarn, 13—Unwinding system.

The electrospinning nanofiber yarn forming device is shown in Figure 1a. The spinning solution was transported to a single needle through a pipe conveying fluid by an injection pump. The polymer formed nanofibers under the action of a high voltage electric field, and orientated nanofibers formed between the grounding disk and the grounding ring. The rotation of the metal disk twists the oriented nanofibers to form nanofiber yarns. The electrospinning nanofiber-wrapped yarn forming device is shown in Figure 1b. Figure 1b has a larger core yarn unwinding system (13) than Figure 1a, which is marked in red. The 75D/36F non-twisted polyester multifilament unwound from the bobbin was used as the core yarn and passed through the center of the disk and the center of the ring, in turn. The rotating metal disk twisted the oriented nanofibers and wrapped the nanofibers on the core yarn. The fiber covered the structural yarn, and the formed covered yarn was finally wound on the finished bobbin by a winding device.

#### 3.2.2. Preparation of Electrospinning Solution

PAN powder with a mass fraction of 12% was added to DMF solvent and stirred at a constant temperature of 40 °C overnight. An electrospinning nanofiber yarn forming device was arranged in accordance with Figure 1. The specific parameters are as follows: the receiving disk has a 10 cm radius and 1 mm thickness, the material was stainless steel, the hole diameter of the middle opening was 1.0 cm; the distances between the plane of the needle tip and disk center were 7 cm, 8 cm, 9 cm, 10 cm, and 11 cm. The inner diameter of the needle tube was 1 mm, the outer diameter was 1.4 mm, the length of the needle tube was 13 mm; the angle between the needle tube and disk was 45°; the center of the ring and disk were horizontal, and the distances between the two were 3 cm, 4 cm, 5 cm, 6 cm, and 7 cm. The ring was made from stainless steel, the outer ring radius was 0.5 cm, the inner ring radius was 0.3 cm, the thickness was 2 mm. The voltage was 18 kV. The flow rate of the pump was 0.6 mL/h, and the disk rotation speed was 40 r/min, 60 r/min, 80 r/min, 100 r/min, and 120 r/min. The spinning time was 15 min, the ambient temperature was 25 °C, and the ambient humidity was 65%.

#### 3.2.3. Analytical Test Method

The surface morphology of the electrospun nanofiber yarn and its wrapped yarn was studied by a JSM-6390 scanning electron microscope (SEM, JEOL, Beijing, China). The voltage was 15 kV, and the magnification was 200, 300, and 5000 times. The SEM images were analyzed using Adobe Acrobat 9 Pro software to calculate the average diameter of yarns and fibers. The prepared yarn was subjected to a tensile testing standard ISO 2062:1993 using an Instron 3365 electronic strength tester (Micoforce, Shanghai, China), and the average was taken from 10 repeated measurements for each sample.

## 4. Experimental Data and Analysis

### 4.1. Electric Field Simulation Analysis of Electrospinning Nanofiber Yarn Forming Device

To theoretically verify the feasibility of electrospinning nanofiber-wrapped structures using a yarn-making device, Maxwell analysis software was used to analyze the electrostatic field to determine the scalar potential distribution of electric fields generated by charge distribution or an applied potential. Because the movement of nanofibers in an electrostatic field is very complex, we simplified the electric field model; therefore, the electric field distribution formed between the nozzle, the receiving ring, and the receiving disk is the main factor to consider in this simulation.

**Figure 2:**
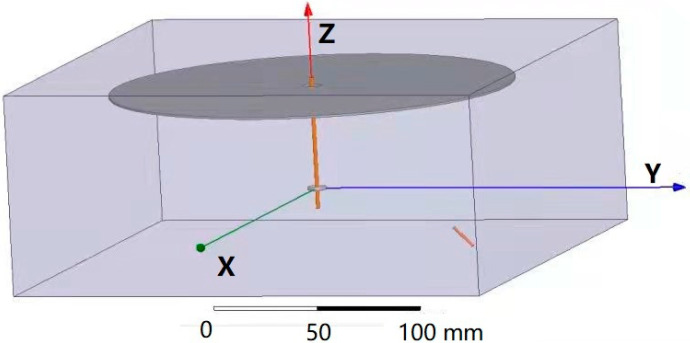
Spatial position model.

The three-dimensional space model established according to the experimental device is shown in Figure 2. The high voltage power supply, bracket, platform, and other devices that have little influence on the electrostatic field were neglected, leaving only the single-needle nozzle and the receiving ring and disk portion, which greatly influence the working electric field. The distance between the ring and disk was set to 9 cm, the angle between the needle and the disk was 45°, the voltage was 18 kV, and the other parameters were the same as in Section 3.2.2. The needle was located in the lower right corner of Figure 2, which was marked as a short brown line.

**Figure 3:**
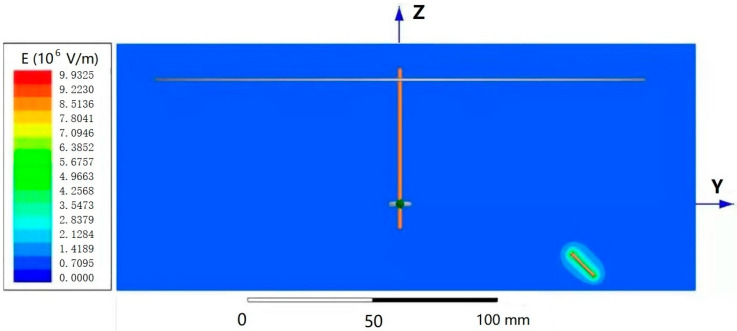
Potential distribution nephogram.

Figure 3 shows the potential distribution nephogram of the electric field and the needle tip portion of the single-needle nanofiber-wrapped structure yarn forming device under an applied voltage of 18 kV. The different colors represent the level of the electric potential, which increases from blue to red. It can be seen that the red area is most concentrated near the needle tip, indicating the highest potential; the blue area near the receiving disk and the ring were also concentrated, indicating the lowest potential. As the distance from the single-needle tip increased, the potential decreased, and the color changed. This verifies the basic principle of electrospinning and verifies the feasibility of the device from a theoretical basis.

**Figure 4:**
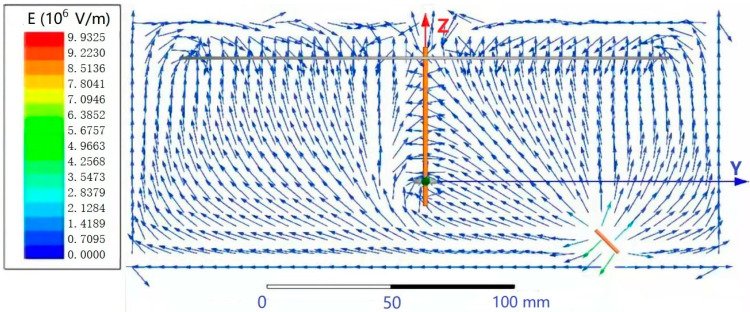
Electric field simulation vector diagram.

As shown in Figure 4, the peak electric field intensity appears near the needle, and the electric field intensity vectors of the needle nearly all pointed to the receiving disk and ring, in accordance with experimental phenomena.

### 4.2. SEM Image Analysis

#### 4.2.1. Effect of Changing the Distance from the Ring to the Disk on the Surface Morphology of the Fiber

**Figure 5:**
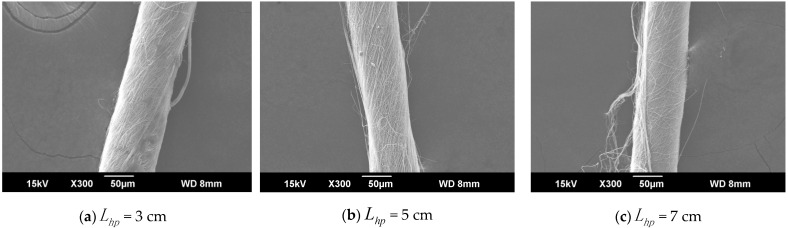
SEM image of nanofiber yarns at different distances between the ring and disk. (**a**) SEM image of nanofiber yarns at $L_{hp}$= 3 cm. (**b**) SEM image of nanofiber yarns at $L_{hp}$ = 5 cm. (**c**) SEM image of nanofiber yarns at $L_{hp}$ = 7 cm.

**Figure 6:**
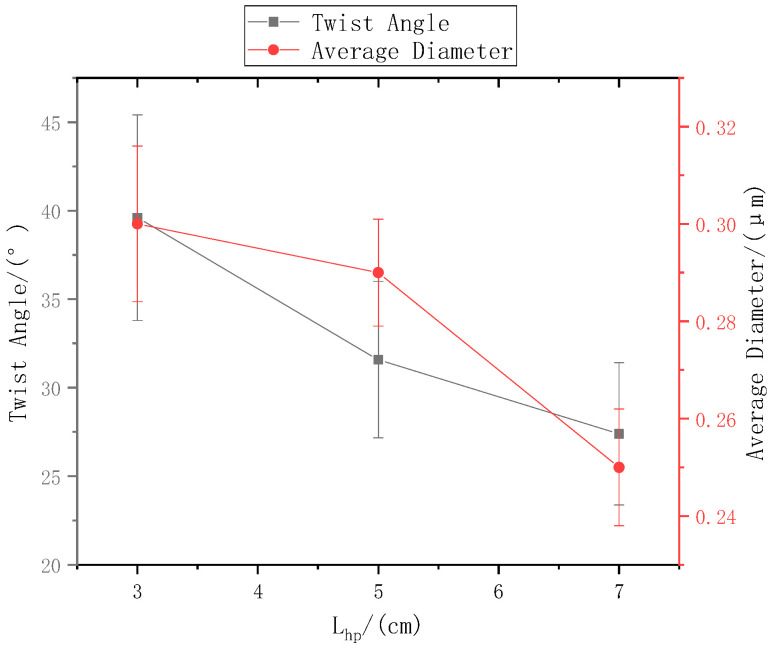
The relationship between the ring-to-disk distance and the nanofiber yarn twist angle and nanofiber diameter.

When the distance from the ring to the disk was set to $L_{hp}$, the distance $L_{zp}$ from the needle to the center of the disk is 9 cm, and the rotation speed n of the disk was 40 r/min. The SEM images in Figure 5a,b, and c respectively show that the nanofiber yarn was prepared by changing $L_{hp}$ from 3 cm, 5 cm, and 7 cm. The nanofibers formed yarn twisted in the z-direction, and the average twist angle decreased as $L_{hp}$ increased (Figure 6). At a constant disk rotation speed, the number of twists per unit time was constant, and the twisting of the nanofiber yarn theoretically remained unchanged. The diameters of the electrospinning nanofiber yarns in a, b, and c of Figure 5 were (88.0 ± 5.3) µm, (77.0 ± 3.9) µm, and (76.8 ± 3.0) µm, respectively. As the distance from the ring to the disk increased, the diameter of nanofiber yarns increased because the greater the twist angle β of the yarn with the same twist, the greater the twist of the fiber, resulting in a thicker yarn. Nanofibers oriented between disks and rings tend to be stretched and refined upon increasing the distance between them.

#### 4.2.2. Effect of Changing the Distance between the Tip of the Needle and the Center of the Disk on the Surface Morphology of the Fiber

**Figure 7:**
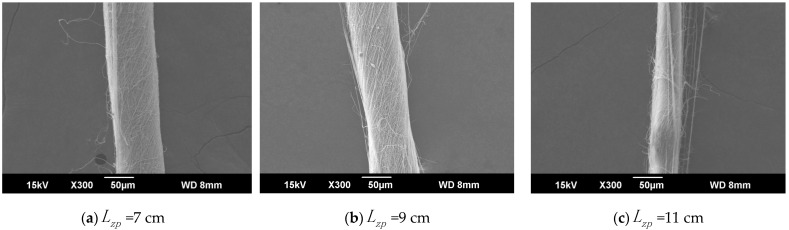
SEM images of nanofiber yarns at different distances between the needle tip and the disk center. (**a**) SEM image of nanofiber yarns at $L_{zp}$ = 7 cm. (**b**) SEM image of nanofiber yarns at $L_{zp}$ = 9 cm. (**c**) SEM image of nanofiber yarns at $L_{zp}$ = 11 cm.

**Figure 8:**
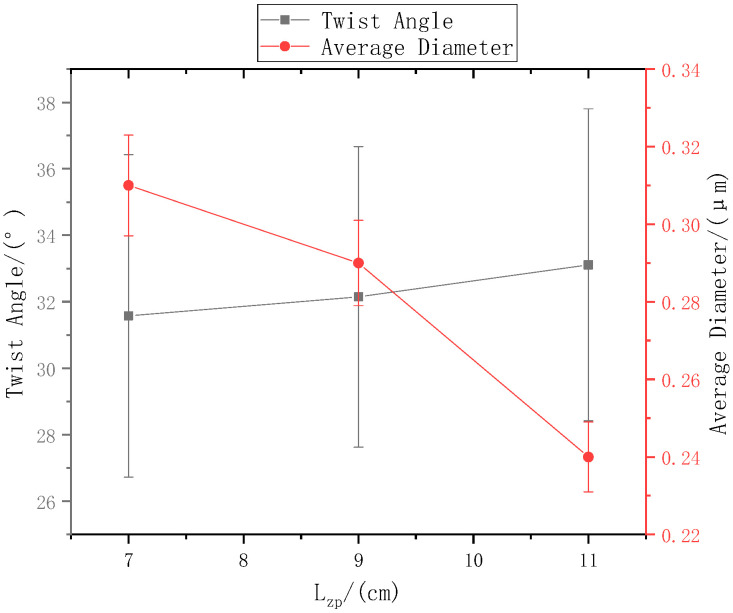
The relationship between the distance from the needle tip to disk center and the nanofiber yarn twist angle and nanofiber diameter.

The ring-to-disk distance $L_{hp}$ was 5 cm, and the rotation speed n of the disk was 40 r/min. The SEM images in Figure 7a, b, and c respectively show the nanofiber yarns prepared by changing $L_{zp}$ at 7 cm, 9 cm, and 11 cm. Figure 7a,b, and c show that the nanofibers formed z-twisted twisted yarns, and the average twist angle of the nanofiber yarns slightly increased upon increasing $L_{zp}$. The difference between the twist angles was not large, but a slight increase was observed in Figure 8. The diameters of nanofiber yarns prepared with $L_{zp}$ = 7 cm, 9 cm, and 11 cm were (80 ± 7.81) µm, (77 ± 3.9) µm, and (48.4 ± 5.43) µm, respectively. The nanofiber yarn diameter decreased farther away from the center of the disk. Fewer oriented nanofibers between the ring and the disk formed when the needle was farther away, and there were more non-oriented fibers; however, less-oriented nanofibers were twisted, resulting in a finer twisted yarn. The farther the needle was from the disk center, the smaller the average diameter of the nanofibers in Figure 8 because at greater distances, the nanofibers had more time to stretch in the electric field, and their average diameter decreased.

#### 4.2.3. Effect of Changing the Disk Rotation Speed on the Fiber Surface Morphology

**Figure 9:**
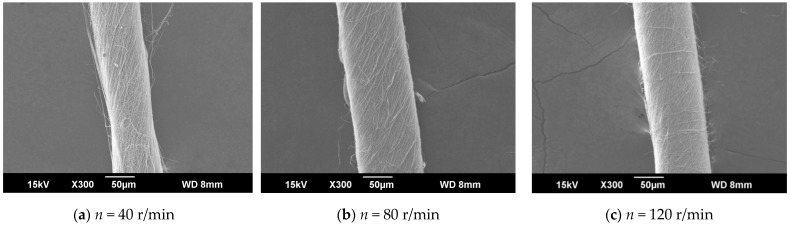
SEM images of nanofiber yarns at different distances between the needle tip and disk center. (**a**) SEM image of nanofiber yarns at *n* = 40 r/min. (**b**) SEM image of nanofiber yarns at *n* = 80 r/min. (**c**) SEM image of nanofiber yarns at *n* = 120 r/min.

**Figure 10:**
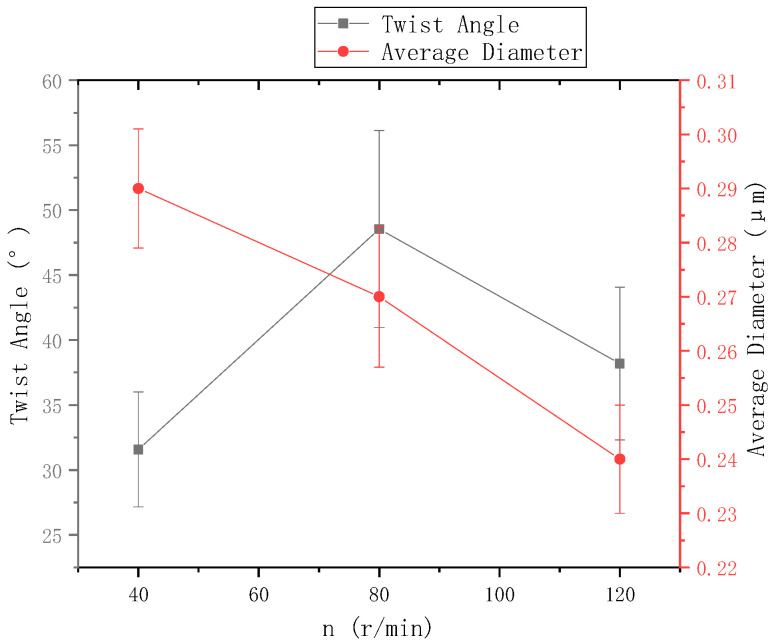
The relationship between disk rotation speed and nanofiber yarn twist angle and nanofiber diameter.

The ring-to-disk distance $L_{hp}$ was 5 cm, and the distance $L_{zp}$ from the needle to the disk center was 9 cm. The images a, b, and c in Figure 9 respectively show the SEM images of nanofiber yarns prepared by changing the rotational speed n of the disk (40 r/min, 80 r/min, and 120 r/min). It can be seen from a, b, and c of Figure 9 that the nanofibers formed a z-twisted twisted yarn, and as the disk rotation speed increased, the average twist angle of the nanofiber yarn first increased and then decreased, but this was not obvious in Figure 10. This feature is consistent with the theoretical analysis below. There is no significant relationship between the twist angle of the nanofiber yarn and the disk rotation speed, only between the ring-to-disk spacing and the position of the nanofibers in the disk. Interestingly, when the disk rotation speed increased, in addition to more closely arranged nanofibers in the nanofiber yarn, some of the nanofibers mutually adhered due to the slow evaporation of the solvent. In a, b, and c of Figure 9, the average nanofiber yarn diameter at disk rotation speeds of 40 r/min, 80 r/min, and 120 r/min were (77 ± 3.9) µm, (106.5 ± 4.83) µm, (93.0 ± 4.36) µm, respectively. There is no obvious change in the average diameters of nanofiber yarns. The results show that in addition to satisfying the basic spinning principles and rules, there are many variables in when electrospinning nanofibers. The average nanofiber diameter decreased upon increasing the disk rotation speed.

#### 4.2.4. Electrospun Nanofiber-Wrapped Structural Yarns after Changing $L_{zp}$

**Figure 11:**
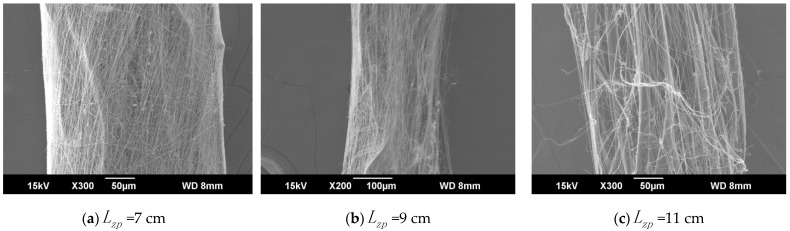
SEM images of electrospun nanofiber-wrapped yarns. (**a**) SEM image of electrospun nanofiber-wrapped yarns at $L_{zp}$ = 7 cm. (**b**) SEM image of electrospun nanofiber-wrapped yarns at $L_{zp}$ = 9 cm. (**c**) SEM image of electrospun nanofiber-wrapped yarns at $L_{zp}$ = 11 cm.

The ring-to-disk distance $L_{hp}$ was 5 cm, the ring-to-disk center penetrated the core yarn of a 75D/36F twistless polyester multifilament, the disk rotation speed n was 80 r/min, and the spinning time was 5 min. The SEM images in Figure 11a–c show the electrospun nanofiber-wrapped structural yarns prepared by changing the distance $L_{zp}$ from the tip to the center of the disk from 7 cm, 9 cm, to 11 cm, respectively. It can be seen from Figure 11a that the nanofiber formed a z-direction twisted yarn with a twist angle of about 19.11°. The twist angle of the nanofiber yarns was 38.19° under the same condition without the core yarn. This confirms the basic yarn theory that the twist angles of the fibers in each layer of the spinning yarns were different. The nanofiber-wrapped structural yarns in Figure 11b,c do not show significant twist directions, and the nanofibers were disordered because $L_{zp}$ was relatively large, and there were fewer oriented nanofibers between the ring and the disk. The spinning time was also shorter, the oriented nanofibers and some of the nanofibers directly injected into the yarns overlapped each other, resulting in a disordered arrangement between the nanofibers. Many oriented nanofibers were also observed in Figure 11b,c, and their alignments were oriented.

Figure 11a–c show that when $L_{zp}$ = 7 cm, the nanofiber-wrapped core yarn was relatively uniform, and no core exposure was observed. However, in b and c of Figure 11, the exposed core was completely visible through the surface of the nanofibers. The core yarn was composed of many untwisted monofilaments. The Figure 11a–c show that at a certain spinning time, as $L_{zp}$ increased, the effect of the nanofiber wrapping structure became increasingly worse, and the wrapping surface became smaller. This is also because, at greater distances, there were fewer oriented nanofibers between the ring and the disk, and the nanofiber yarn diameter decreased as $L_{zp}$ increased. However, we can also infer that as the spinning time of the electrospun nanofiber-wrapped structure yarns was extended from 5 min to 15 min, more oriented nanofibers were wrapped on the core yarn, and the wrapping effect was better.

It can also be seen from Figure 11a–c that the nanofibers were arranged parallel and adhered to each other. This phenomenon confirms that the individual oriented nanofibers were pushed together during twisting and bonded as the solvent evaporated. In panels Figure 11a–c, for electrospun nanofiber-wrapped structural yarns prepared at $L_{zp}$ = 7 cm, 9 cm, and 11 cm, the corresponding nanofibers have an average diameter of 0.28 µm, 0.26 µm, and 0.25 µm, respectively. As $L_{zp}$ increased, the average nanofiber diameter also decreased.

### 4.3. Mechanical Performance Analysis

The mechanical tests return the breaking strength, which must be converted into the fracture strength as follows σ = Fb/So. The cross-sectional area of the sample was measured from the SEM images, and the diameter is converted according to the area formula.

#### 4.3.1. Change the Electrospinning Nanofiber Yarn under $L_{zp}$

**Figure 12:**
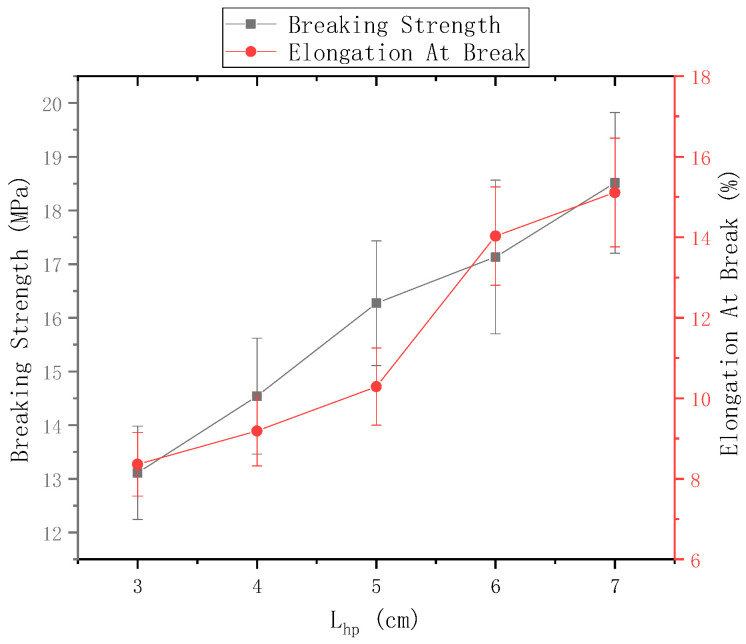
Mechanical properties of nanofiber yarns at different distances between the ring and the disk.

The distance $L_{zp}$ of the needle from the center of the disk was 9 cm and the rotational speed n of the disk was 40 r/min. Figure 12 shows the mechanical properties of electrospun nanofiber yarns prepared using ring-to-disk distances $L_{hp}$ of 3 cm, 4 cm, 5 cm, 6 cm, and 7 cm. Figure 11 shows that the breaking strength of nanofiber yarns prepared was the highest (18.51 ± 1.31) MPa, when $L_{hp}$ = 7 cm, and the elongation at break was the highest 15.11%, which is more suitable for traditional weaving processing. The fracture strength and elongation at break of the nanofiber yarns increased gradually, mainly because the length of the oriented nanofibers between the rings and disks increased upon increasing the distance, which strengthened the orientation degree of macromolecules along the nanofiber direction, and thus improved the mechanical properties of the fibers. Theoretically, if the distance between the needle tip and the center of the disk exceeds a certain limit, few nanofibers will be collected by the device due to the large receiving distance, and yarn will not form.

**Figure 13:**
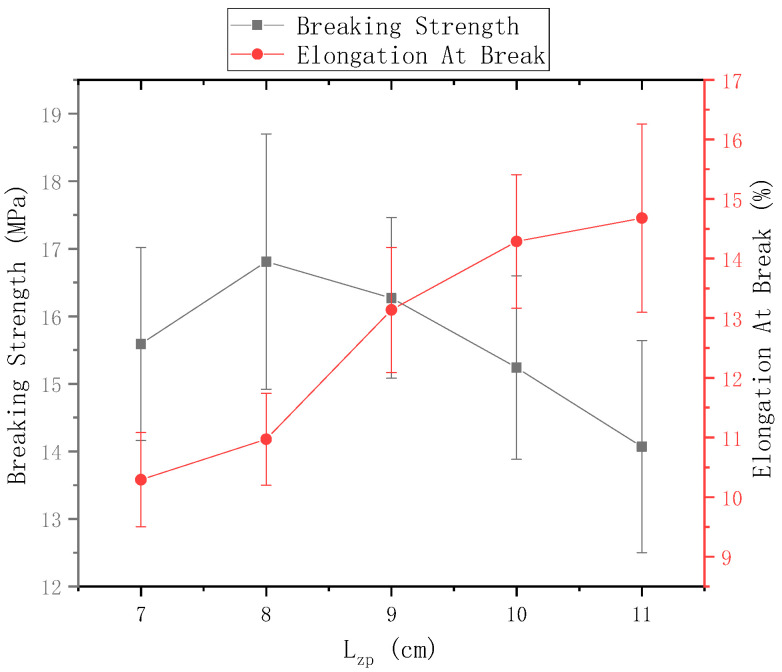
Mechanical properties of nanofiber yarns at different distances between the needle tip and disk center.

The ring-to-disk distance $L_{hp}$ = 5 cm, and the rotational speed n of the disk is 40 r/min. Figure 13 shows the mechanical properties of electrospun nanofiber yarns prepared when the distance between needle and disk center $L_{zp}$ = 7 cm, 8 cm, 9 cm, 10 cm, and 11 cm. The breaking strength of the nanofiber yarn was the highest (16.81 ± 1.89) MPa, when $L_{zp}$ = 8 cm, but its breaking elongation did not reach a maximum. As $L_{zp}$ between the needle and the disk center increased, the fracture strength of the yarn increased first and then decreased, and the elongation at break always increased. As $L_{zp}$ increased, the nanofibers had sufficient time to stretch and draw in the electric field, and the macromolecular orientation in the nanofibers was consistent with the axial direction, so that the fracture strength of the yarn increased; however, when $L_{zp}$ was large, some nanofibers were scattered outside the twisted area, and no oriented nanofibers formed between the disk and the ring. Thus, too few fibers were formed in the nanofiber yarn, which decreased the fracture strength.

**Figure 14:**
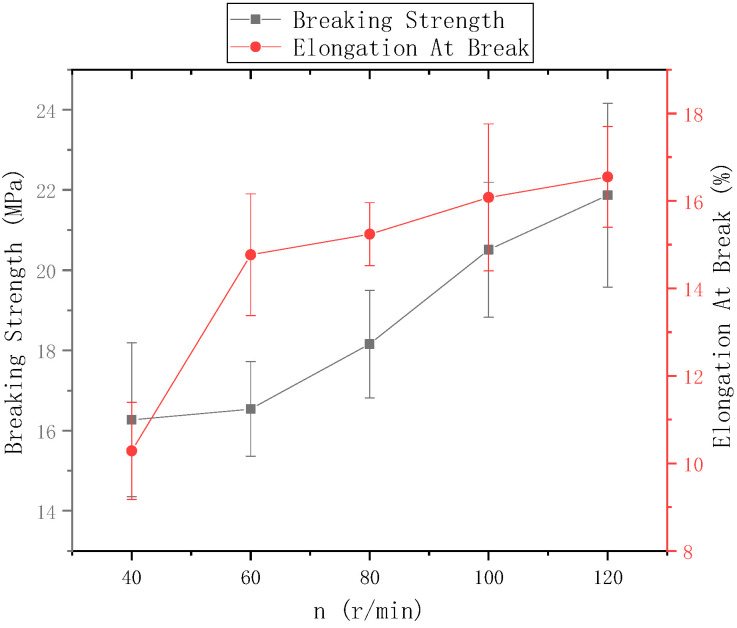
Mechanical properties of nanofiber yarns at different disk speeds.

The ring-to-disk distance $L_{hp}$ = 5 cm and the distance $L_{zp}$ between the needle and the center of the disk was 9 cm. Figure 14 shows the mechanical properties of electrospun nanofiber yarns prepared when the rotational speed n of the disk was 40 r/min, 60 r/min, 80 r/min, 100 r/min and 120 r/min. Figure 13 shows that the highest breakage strength of nanofiber yarns prepared at n = 120 r/min was (21.87 ± 2.29) MPa, and the highest elongation at break was 16.55%. The fracture strength and elongation at break of nanofiber yarns tended to gradually improve at a disk rotation speed of 120 r/min or lower. The rotating metal disk twisted the oriented nanofibers. Increasing the disk rotation speed increased the degree of nanofiber twisting, and the bonding between the nanofibers tightened, which increased the fracture strength and elongation at break of the nanofiber yarns. As the disk rotation speed increased, the mechanical properties of the nanofiber yarns did not always improve. Theoretically, there is a limit, and when this limit is exceeded, the mechanical properties will decrease. Because the rotation speed was too high, the nanofibers broke, and a yarn did not form.

#### 4.3.2. Electrospun Nanofiber-Wrapped Structural Yarns after Changing $L_{zp}$

**Figure 15:**
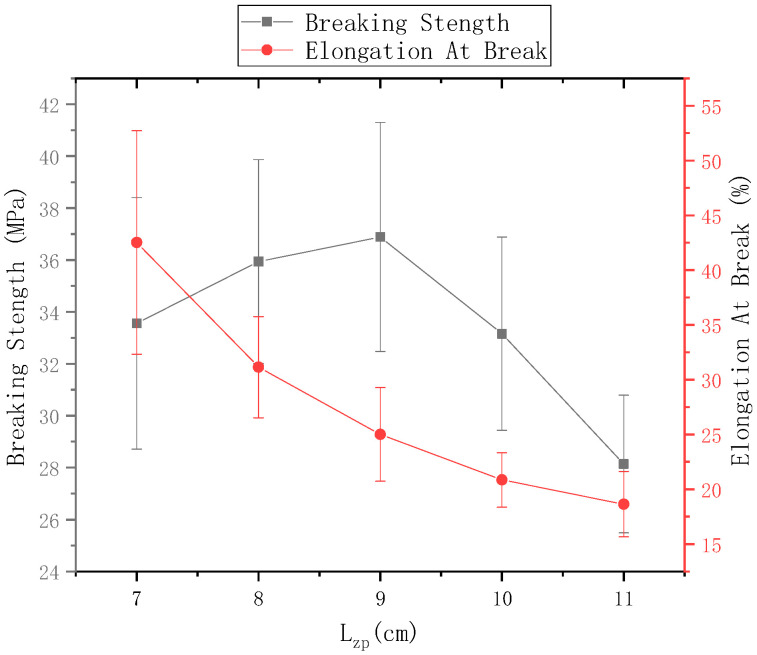
Mechanical properties of nanofiber-wrapped yarns at different distances from the tip of the needle to the disk center.

The ring-to-disk distance $L_{hp}$ = 5 cm, the ring-to-disk center penetrated the core yarn of the 75D/36F twistless polyester multifilament, the disk rotation speed n = 80 r/min, and the spinning time was 5 min. The fracture strength of electrospun nanofiber-wrapped structural yarns prepared at c $L_{zp}$ = 7 cm, 8 cm, 9 cm, 10 cm, and 11 cm are shown in Figure 15, and the elongation at break is shown in Figure 14. It can be seen from Figure 15 that as the distance from the tip of the needle to the center of the disk increased, the fracture strength first increased and then decreased. The elongation at break decreased, but the fracture strength and elongation at break of the nanofiber-wrapped yarns were substantially higher than those of the core yarn. The reason is that the oriented nanofibers were wrapped on the surface of the core yarn by twisting, and the multifilaments in the core yarn increased due to friction and cohesion. These nanofibers were basically submicron fibers. The twisted-oriented nanofibers enhanced the core yarn. After the twistless yarns broke, the wrapped nanofibers that were not pulled off outside the core yarn continued to stretch; therefore, the fracture strength and elongation at break of the yarns after wrapping with nanofiber were improved. However, when the distance from the tip of the needle to the disk center was 11 cm, there were fewer oriented nanofibers between the ring and the disk, which led to a low coverage rate of the nanofiber-wrapped yarns. The yarn strength and elongation at break were much lower than when $L_{zp}$ = 9 cm.

## 5. The Nanofiber-Wrapped Yarn Formation Mechanism

**Figure 16:**
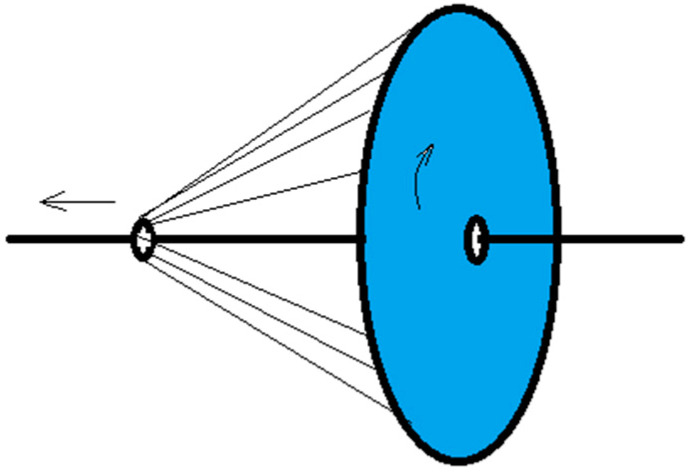
Schematic diagram of the formation of electrospun nanofiber-wrapped yarns.

The formation of electrospun nanofiber-wrapped structural yarns occurs via the formation of many oriented nanofibers between the disk and the ring. The rotation of the disk drives the oriented nanofibers to coat the core yarn at an angle. Its schematic diagram is shown in Figure 16.

### 5.1. Wrapping Mechanism under Specific Assumptions

It is assumed that the ring aperture is exactly the same as the diameter of the core yarn, and the inner and outer diameters of the ring are the same. After this simplification, according to the electric field nephogram in Figure 3, one of the two ends of the oriented nanofibers is on the ring, and the other end is at any point on the disk. The distance from the endpoint to the center of the disk is d_0_ (mm), and the length of the oriented nanofibers is $L_{q}$ (mm). At the same time, the core yarn may be wound up at a winding speed of *v* (mm/s). The twist angle is *β* (°), the disk rotation speed is *n* (r/min), the core yarn is assumed to be a cylinder with a diameter d (mm), the ring diameter is *D*_1_ (mm), the disk diameter is *D*_2_ (mm), and the disk aperture is *D*_0_ (mm), and the twisting time is *t* (s).

**Figure 17:**
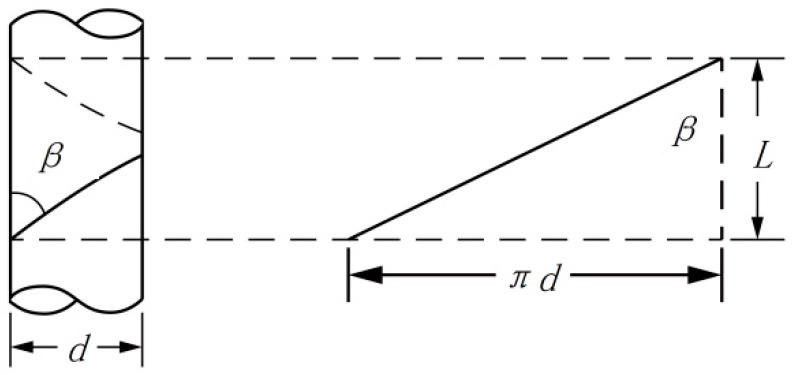
Schematic diagram of the nanofibers twisted on the core yarn.

It is assumed that oriented nanofibers were formed at a certain fixed position between the ring and the disk and that they do not break during twisting. The twisted nanofibers are inclined, and the greater the twisting degree, the greater the incline. Therefore, the inclination angle of the nanofibers on the core yarn—twist angle *β*, i.e., the angle between the nanofiber surface and the yarn axis (Figure 17), can be calculated by the following Formula (1):

(1) $$\tan\beta =\frac{\pi d}{L}$$

In the formula, *β* is the twist angle (°), d is the core yarn diameter (mm), and L is the nanofiber displacement along the core yarn axis when twisted at a fixed angle, *β*. At this time,

(2) $$L=\frac{100}{T_{\mathrm{tex}}}$$

In the formula, $T_{\mathrm{tex}}$ is the twist of nanofibers, twist/10 cm.

The distance the core yarn moves in the axial direction in a twist, i.e., the distance the core yarn is wound during a twist is *λ*.

(3) $$\lambda =v\frac{L}{\frac{n}{60}}=\frac{60vL}{n}$$

In the formula, *v* is the core yarn winding speed, and *n* is the disk rotation speed.

The displacement difference *A* of the core yarn wound in one twist turn is the difference between the core yarn winding distance *λ* in one twist turn and the displacement *L* of the nanofiber moving along the axial direction of the core yarn in one twist turn.

(4) A=λ−L

*A* can adopt one of three different values: *A* = 0, *A* > 0, and *A* < 0.

(1) *A* = 0,

$$\lambda =L=\frac{60vL}{n}\Rightarrow v=\frac{n}{60}$$

The winding speed is equal to the displacement of nanofibers moving along the axial direction of the core yarn in a given twisting time, i.e., the starting point of each twist is the same. At this time,

(5) $$\tan\beta =\frac{\pi d}{L}=\frac{\pi d}{v\frac{n}{60}}=\frac{60\pi d}{vn}$$

**Figure 18:**
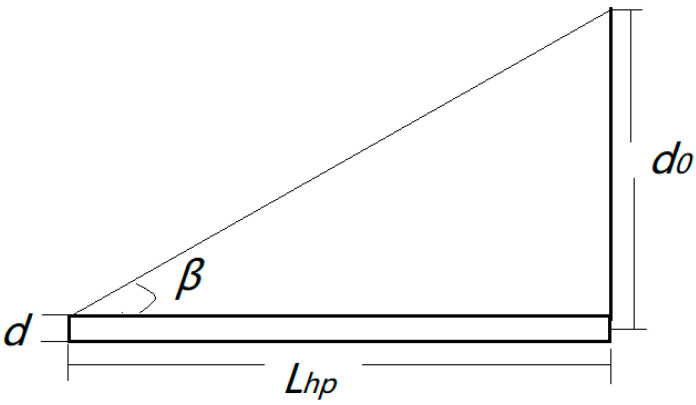
Schematic diagram of disk twisting.

A schematic diagram of the disk twisting is shown in Figure 18, where

(6) $$\tan\beta =\frac{d_{0}-\frac{d}{2}}{L_{hp}}$$

In this case, the twist angle *β* is constant, which can be applied to the traditional twist coefficient Formula (7).

(7) $$tg\beta =\frac{\pi dT_{tex}}{100}$$

The twisting of the nanofiber yarns on a cylindrical core yarn is somewhat similar to winding the yarns on a cylindrical bobbin at a constant winding angle. The difference between the two is that winding yarns is continuous because the yarns can be continuously supplied, while oriented nanofibers twisted on a core yarn are discontinuous. Oriented nanofibers are formed between the ring and the disk. During twisting, the oriented nanofibers are wrapped on the core yarn, while the two ends of the oriented nanofibers remain between the ring and the disk. The twist angle of the same oriented nanofiber remains constant when twisted. The greater the twist angle β of the yarns with the same twist, the greater the twist of the fibers and the thicker the yarns. Figure 5 and Figure 6 verify Formula (7) and explain its trend.

Some researchers have used the edge of a disk to collect oriented nanofibers and then used the disk to twist the nanofibers to form yarns. By increasing the rotational speed of the disk, the twist angle increased accordingly. This study also verified Formulas (5) and (7) [32].

The length of the wrapping of each twisted nanofibers is given by:

(8) $$L_{0}=\frac{L}{\cos\beta }=\frac{\pi d}{\sin\beta }$$

when *L*_0_ > *L*, the oriented nanofibers are stretched during twisting. It is assumed that the elongation at break of the nanofibers is ε, the value of which depends on the nature of the polymer used in the electrospinning solution and the electrospinning process parameters.

When the oriented nanofibers are twisted, the number of twists is m, and the length of the oriented nanofibers is *L*_q_, then:

$$mL_{0}>L_{q}(1+\varepsilon ),\;i.e.,\;m\frac{L}{\cos\beta }=m\frac{\pi d}{\sin\beta }>L_{q}(1+\varepsilon )$$

At this time, the oriented nanofibers will break, and they will no longer be twisted on the core yarn by a twist angle *β*. The nanofibers will wrap on the core yarn with the oriented nanofibers irregularly formed behind them during twisting. Therefore, only some of the oriented nanofibers that are twisted on the core yarn form a stable twist angle *β*, and most irregularly wrap on the core yarn. Some nanofibers are directly deposited on the core yarn, and they are also irregular. These inferences are consistent with the experimental phenomena in this paper. There are many oriented nanofibers on the same layer that are irregularly wrapped on the core yarn. The phenomenon in which some nanofibers are twisted on the yarns in an irregular form in Figure 8a, b and c also verifies these laws.

(2) *A* > 0

Here, there are two situations. The first is $L<\lambda \leq L_{q}(1+\varepsilon )\cos\beta$, and the second one is $\lambda >L_{q}(1+\varepsilon )\cos\beta$.

When $L<\lambda \leq L_{q}(1+\varepsilon )\cos\beta$, the corresponding phenomenon and conclusion are the same as *A* = 0.

When $\lambda >L_{q}(1+\varepsilon )\cos\beta$, the nanofibers that are not twisted are broken by the high-speed winding core yarn during twisting, so the nanofiber-wrapped structural yarns cannot form.

In the above two situations where *A* > 0, it is assumed that the twist angle is fixed. The actual situation is that the twist angle changes, and the twist angle *β*’ has the following characteristics:

(9) $$\tan\beta \prime =\frac{d_{0}-\frac{d}{2}}{L_{\mathrm{hp}}-vt}$$

Formula (10) shows that the twist angle *β*’ changes continuously over time, and the twist angle *β*’ constantly increases. The maximum twist angle *β*_0_’ has the following formula:

(10) $$\tan\beta_{0}^{\prime }=\frac{d_{0}-\frac{d}{2}}{L_{\mathrm{hp}}-\int_{0}^{\frac{60m}{n}}vdt}$$

(3) *A* < 0

During a twist,

(11) $$\tan\beta \prime \prime =\frac{\pi \pi d}{L}$$

At this time, according to the disk twisting diagram in Figure 16:

(12) $$\tan\beta \prime \prime =\frac{d_{0}-d}{L_{hp}-\sum_{1}^{n}\left(L_{x}-\lambda_{x}\right)}$$

In the formula, x is the xth twist.

Formula (12) shows that *β*” is a variable, and as the number of twists increases, the twist angle becomes larger. During a twist, *L* in Formula (12) becomes smaller as the number of twists is increased.

Formula (8) shows that the wrapping length formula of each twisted nanofiber is as follows.

(13) $$L_{0}=\frac{L}{\cos\beta \prime \prime }=\frac{\pi d}{\sin\beta \prime \prime }$$

When oriented nanofibers are twisted, the twisting number is m, and the length of the oriented nanofibers is *L*_q_, then

$$mL_{0}>L_{q}(1+\varepsilon ),\;i.e.,\;m\frac{L}{\cos\beta \prime \prime }=m\frac{\pi d}{\sin\beta \prime \prime }>L_{q}(1+\varepsilon )$$

At this time, the oriented nanofibers will break, and the nanofibers are no longer twisted on the core yarn by a twist angle, *β*′. The nanofibers will wrap on the core yarn with the oriented nanofibers irregularly formed behind them during twisting. Therefore, only some of the oriented nanofibers that are twisted on the core yarn will form a stable twist angle *β*, and most will wrap on the core yarn in an irregular form. Some nanofibers will directly deposit on the core yarn, and this part of the nanofibers is also irregular. However, when the fiber head end is twisted to a certain degree, the nanofibers will be pulled out from the grip of the disk and twisted into the yarn body by the next layer of nanofibers in an irregular shape.

### 5.2. Wrapping Mechanism under Non-Specific Conditions

#### 5.2.1. Inner and Outer Diameters of the Ring Are Not Ignored

**Figure 19:**
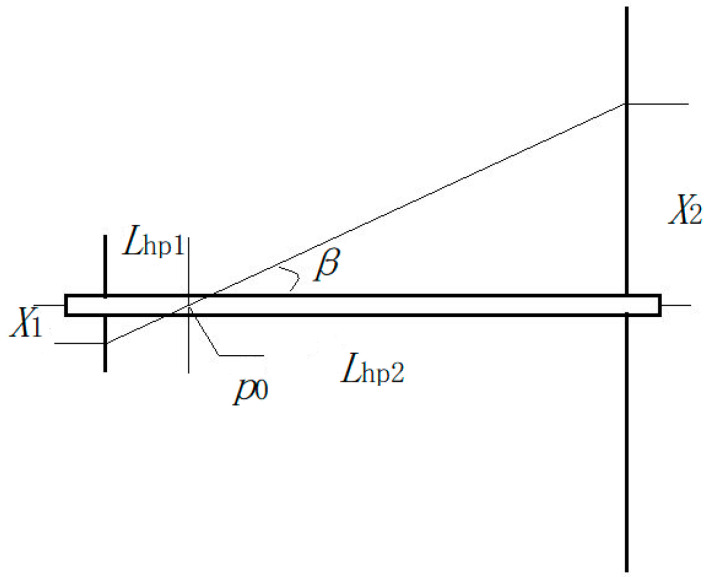
Oriented nanofiber twisting schematic.

All the above inferences and conclusions are based on the assumption that the ring aperture and the core yarn diameter are identical, as are the inner and outer diameters of the ring. The actual situation is that the ring aperture and the core yarn diameter are different, and the inner diameters and the outer diameter of the ring are also different. According to the electric field vector diagram in Figure 4, one of the two ends of the oriented nanofibers may be located at any point between the inner and outer diameters of the ring, and the other end may be located at any point on the non-central hole of the disk. The distance between the nanofiber at one end of the ring and the center of the core yarn is x_1_, and the distance between the end of the disk and the center of the core yarn is x_2_. The twisting of oriented nanofibers is shown in Figure 19.

When the oriented nanofiber is twisted, it will always contact the core yarn, and when it is twisted further, it will wrap the core yarn at a twist angle *β*. The position at which the twisting begins is shown by p0 in Figure 19. The twisting start position is described as follows:

(14) $$\frac{X_{1}}{X_{2}}=\frac{L_{\mathrm{hp}1}}{L_{\mathrm{hp}2}}$$

(15) $$L_{\mathrm{hp}1}+L_{\mathrm{hp}2}=L_{\mathrm{hp}}$$

In the $L_{\mathrm{hp}1}$ segment, since the ring is not rotated, twisting does not occur. The principle of twisting in the $L_{\mathrm{hp}2}$ segment is the same, in which it is assumed that the ring diameter and core yarn diameter are identical, and the inner and the outer diameters of the ring are also the same; therefore, the inferences and conclusions apply equally.

#### 5.2.2. Randomly Fixed Nanofiber ends

The above inferences and conclusions are based on the assumption that oriented nanofibers are formed at a fixed position between the ring and the disk, i.e., the environment in which oriented nanofibers is fixed. The actual situation is relatively complicated. One of the two ends of an oriented nanofiber may be located at any point between the inner and outer diameters of the ring, while the other end can be at any point on the non-central hole of the disk. The twisting process mainly involves disk twisting, so only the position of the nanofibers on the disk affects the twist angle.

The distance *x*_i_ between the nanofibers at the disk and the center of the core yarn is a random variable, which can be a finite number or infinity and can be listed one by one, and its twist angle is one or several finite or infinite intervals. This random variable $x_{i}$ is a typical discrete random variable. The probability corresponding to *x*_i_ is *p*(*x*_i_), and the sum of the products is the mathematical expectation of the discrete random variable *x*_i_, denoted as E(*x*).

The value of the discrete random variable *x* is:

$x_{1}$, $x_{2}$, $x_{3}$, …, $x_{n}$, $p\left(x_{1}\right)$,$p\left(x_{2}\right)$,$p\left(x_{3}\right)$,...,$p\left(x_{n}\right)$ the probability that *x* corresponds to the value, then:

$$E\left(x\right)=x_{1}*p\left(x_{1}\right)+x_{2}*p\left(x_{2}\right)+x_{3}*p\left(x_{3}\right)+\ldots +x_{n}*p\left(x_{n}\right)=\sum_{k=1}^{\infty }x_{k}p_{k}$$

In other words, the mathematical expectation $E\left(x\right)$ of the random variable of the distance between the nanofibers on the disk and the center of the core yarn can be used instead of d_0_ in Figure 18 under the assumption that oriented nanofibers are formed at a fixed displacement between the ring and disk. The reasoning and formula are the same. Because of the random variables, there is no obvious change in the twist angles. In Figure 5, Figure 7 and Figure 9, the twist angles of nanofibers in the same layer are not constant, large or small, which verifies the randomness of this phenomenon. The average twist angles of nanofibers in each layer also verifies the existence of this mathematical expectation law.

#### 5.2.3. Coreless Yarn

The above inference is also applicable in the case of a coreless yarn, i.e., in the case where the core yarn diameter d = 0 (Figure 18). At this time, d_0_ in Figure 18 is the distance from the fiber endpoint on the disk to the center of the disk.

Formula (6) becomes $\tan\beta =\frac{d_{0}}{L_{\mathrm{hp}}}$, Formula (8) becomes $L_{0}=\frac{L}{\cos\beta }$, Formula (9) becomes $\tan\beta^{\prime }=\frac{d_{0}}{L_{\mathrm{hp}}-vt}$, Formula (10) becomes $\tan\beta_{0}^{\prime }=\frac{d_{0}}{L_{\mathrm{hp}}-\int_{0}^{\frac{60m}{n}}vdt}$, Formula (13) becomes $L_{0}=\frac{L}{\cos\beta \prime \prime }$.

### 5.3. Analysis of the Uniqueness of the Electrospinning Nanofiber Wrapping Process

#### 5.3.1. Dispersion of Oriented Nanofiber Collection Process

**Figure 20:**
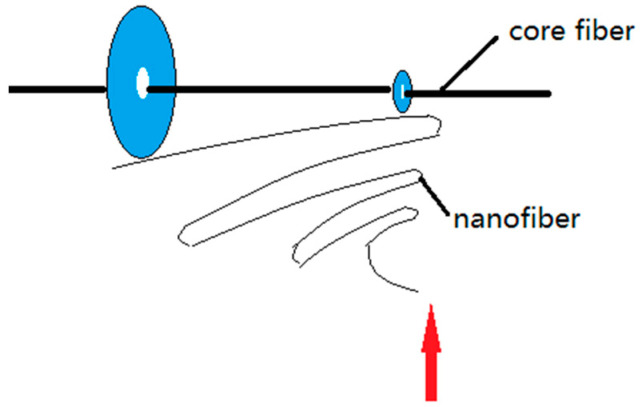
Schematic diagram showing the formation of oriented nanofibers.

Electrospun nanofibers move spirally in an electric field, and their trajectory continually changes. The collection of oriented nanofibers between the ring and the disk is guided by the electric field of the grounding ring and the disk, and oriented nanofibers are deposited between the ring and the disk. These oriented nanofibers are only some of the fibers formed by electrospinning (Figure 20). Some nanofibers escape directly into other non-collecting device areas, and a small portion of the nanofibers are directly sprayed onto the core yarn due to probability. This is caused by the uncertainty of the nanofiber movement during electrospinning when the electric field is stretched. Although the electric field of the grounding ring and the disk will form oriented nanofibers between them, the nanofibers are easily affected by the surrounding environment and their intermolecular forces during stretching in the electric field; thus, their motion trajectories are unpredictable. Some fibers that are stretched out in the electric field escape from the ring and the disk receiving electrode. They escape outside of the receiving range and become flying flowers. This is not the same principle of macro-spinning methods, such as melt spinning. Although all involve polymer stretching, fibers produced by melt spinning are always precisely controlled because both ends are fixed during stretching; however, electrospinning stretching only involves limited guidance from the electrodes, machinery, and fluids.

#### 5.3.2. Discontinuity of Fiber Length Direction during Mechanical Twisting

Figure 20 shows a schematic diagram of the mechanical twisting principle. Oriented nanofibers formed between the disk and the ring are wrapped on the core yarn at an angle with the disk rotation. It can be seen from Figure 20 that the formation of oriented nanofibers is intermittent, and the formation of the first oriented nanofiber is followed by the formation of the second oriented nanofibers, and so on. During twisting, the first nanofiber is formed, and the second nanofiber is formed after the disk is rotated for a time (*t*), and the angle φ between the first and second nanofibers follows the Formula (16):

(16) φ=ωt

where ω is the disk rotation speed.

Formula (18) shows that oriented nanofibers have intermittent formation, but also have a certain angular relationship with each other during mechanical twisting. The discontinuity of fiber length direction is caused not only by the intermittent formation of oriented nanofibers, but also by nanofiber breakage described by the formula:$mL_{0}>L_{q}(1+\varepsilon )$.

The mechanical twisting and wrapping process is obviously different from the wrapped structure, in which the outer wrapping yarn is generally continuously wrapped around the core yarn under the action of machinery and fluid. However, the length of the nanofibers during mechanical twisting wrapping herein is discontinuous.

#### 5.3.3. Non-Uniformity of the Twist Angles during Mechanical Twisting and Wrapping Process

The analysis in Section 5.1 and Section 5.2 showed that the twist angle remained constant during twisting only under certain conditions. In the first case where *A* > 0, *A* = 0, the nanofibers are not broken and are fixed in a certain layer in the yarns. The oriented nanofibers are formed at a fixed position between the disk and the ring. This constant twist angle under only certain conditions is identical to the traditional spinning process. Nanofibers have different twist angles in different yarn layers, which is consistent with the characteristics of traditional spinning.

In the second case where *A* > 0 (Section 5.1), the nanofibers not twisted by the disk rotation are broken by the core yarn. In this case, a twisted wrapping is not formed. In other cases, the nanofibers can be wrapped after being broken. Under this condition, the other end of the broken nanofibers is not held but fluctuates irregularly with the surrounding environment (airflow, electric field, inertia force, and friction force, etc.). Figure 20 is a schematic diagram of nanofibers wrapped on the core yarn after breaking. The red line is the previous layer fiber, and the green line is a subsequent layer fiber. The broken fiber near the end of the disk becomes a flying flower and no longer participates in the twisting wrapping. After breaking, some fibers near the end of the ring fraying outside the yarns, and some are twisted into yarns by the next layer of nanofibers, and the structure of this part of nanofibers is disordered.

According to Section 5.2 and the electric field simulation analysis, one of the two ends of the oriented nanofibers may be located at any point between the inner and outer diameters of the ring, and the other end may be at any point of the non-central hole of the disk. This rule was confirmed in the actual process of wrapping and twisting the yarns. Therefore, from the twist angle calculation Formulas (1), (5), and (7), we can see that the twist angle of nanofibers is not constant, even within the same layer, and there is certain randomness during twisting and wrapping of the disk and ring. The random expectation E(*x*) can be introduced to represent the average twist angle.

## 6. Conclusions

In this paper, a self-made device was used to form oriented nanofibers between a disk and ring. By rotating the disk, the oriented nanofibers were twisted into yarns with a specific angle, and electrospun nanofiber yarns and its multiscale wrapping yarns were prepared. The electric field simulation analysis of the electrospinning nanofiber yarn forming was in agreement with experimental observations. During the preparation of electrospun nanofiber yarns, the average twist angle and diameter of the nanofiber yarns was correlated with changes in the ring-to-disk distance, the tip and the center of the disk, and the disk rotation speed. When preparing electrospun nanofiber-wrapped yarns, oriented nanofibers were arranged in a specific direction. This paper also discussed the mechanism of the nanofiber-wrapped yarn in three cases, according to the displacement difference *A* of the core yarn within one twist. When *A* = 0, the starting point of each twist was the same. When the oriented nanofiber was twisted to a certain degree, it broke, or the fiber tip was pulled out and was no longer held. The nanofibers no longer twisted on the core yarn at a twist angle β, and they irregularly wrapped the core yarn by the latter layer. Compared with traditional spinning processes, the electrospinning nanofiber wrapping process has unique properties, such as the uncertainty of the oriented nanofiber collection process, the discontinuity of the fiber lengthwise direction during twisting, and the non-uniform twist angle. The research conclusion of this paper has guiding significance for accurately controlling the mass production of electrospun micro- and nanofibers.

In this paper, the 24-h output of pure electrospun yarn was about 672 cm, and the output of electrospun micro/nanocore spun yarn was about 2016 cm. It also has certain industrial application value for high value-added tissue engineering, sensors, and other fields. If the single needle is replaced by multiple needled or needle-free electrospinning technology, the output will be much higher than that of traditional single-needle electrospinning. The diameter distribution and yarn strength with the *CV*% were not analyzed because of software limitations. This paper only prepared micro/nanocore spun yarn using ordinary yarn as the inner layer and nanofiber as the outer layer, and it did not study the fabric prepared by this micro/nano-structured yarn or the functionality of this fabric. The research team will carry out further research concerning these limitations. Interestingly, we are studying the electrospun nanofiber-core spun yarn wrapped with a layer of ordinary yarn. This sandwich yarn will protect the nanofibers in the middle layer, which can be processed by traditional weaving technology; thus, they will have broader potential applications in areas such as supercapacitors and functional textiles.
